# Association between Serum Zinc Levels and Clinical Index or the Body Composition in Incident Hemodialysis Patients

**DOI:** 10.3390/nu12103187

**Published:** 2020-10-19

**Authors:** Tatsunori Toida, Reiko Toida, Shou Ebihara, Risa Takahashi, Hiroyuki Komatsu, Shigehiro Uezono, Yuji Sato, Shouichi Fujimoto

**Affiliations:** 1 Department of Hemovascular Medicine and Artificial Organs, Faculty of Medicine, University of Miyazaki, Miyazaki 889-1692, Japan; fujimos@med.miyazaki-u.ac.jp; 2 Department of Internal Medicine, Miyazaki Prefectural Nobeoka Hospital, Nobeoka 882-0835, Japan; risa_takahashi@med.miyazaki-u.ac.jp; 3 Department of Internal Medicine, Chiyoda Hospital, Hyuga 883-0064, Japan; reiko_toida@med.miyazaki-u.ac.jp (R.T.); suezono@pref-hp.miyazaki.miyazaki.jp (S.U.); 4 Division of Circulatory and Body Fluid Regulation, Department of Internal Medicine, University of Miyazaki, Miyazaki 889-1692, Japan; sho_ebihara@med.miyazaki-u.ac.jp; 5 Center for Medical Education and Career Development, Faculty of Medicine, University of Miyazaki, Miyazaki 889-1692, Japan; hiroyuki_komatsu@med.miyazaki-u.ac.jp; 6 Division of Nephrology, Department of Internal Medicine, National Health Insurance Takachiho Town Hospital, Takachiho 882-1101, Japan; ysato@med.miyazaki-u.ac.jp

**Keywords:** serum zinc levels, hemodialysis, nutritional status

## Abstract

Background: The relationships between serum zinc levels and body composition or clinical outcomes of incident hemodialysis (HD) patients remain unclear. Methods: This prospective observational study examined the relationships between serum zinc levels and clinical indexes, including body composition, in 142 incident HD patients using a bioelectrical impedance analysis. Patients were divided into three groups according to baseline serum zinc levels: tertile, <45, 45–59, and ≥60 µg/dL. The reference group was set as ≥60 µg/dL. Cox’s regression analysis was performed to investigate the relationships between serum zinc categories and cardiovascular events and all-cause mortality after adjustments for potential confounders. Results: Serum zinc levels positively correlated with the nutritional index and negatively correlated with fluid volume markers. In a mean follow-up of 2.5 years, there were 20 cases of cardiovascular events and 15 of all-cause mortality. In the Cox’s regression analysis for cardiovascular events and all-cause mortality, the hazard ratio increased with a decrease in serum zinc levels, but was not significant. Conclusion: Serum zinc levels were associated with nutritional and fluid volume markers in incident HD patients. To clarify the relationship between serum zinc levels and cardiovascular events or mortality, further studies with a larger number of cases will be necessary.

## 1. Introduction

Zinc is the second most abundant transition metal in the body after iron and is an essential trace element that plays a crucial role in cell metabolism, growth, tissue repair, the production of neurotransmitters, and antioxidant defenses [1,2,3]. It is also involved in arterial pressure regulation and the etiopathogenesis of arterial hypertension via the renin-angiotensin-aldosterone system [4]. Therefore, zinc deficiency may result in apoptosis, oxidative stress, inflammation, and poorly controlled blood pressure, all of which are risk factors for cardiovascular disease (CVD) [5]. Approximately 60–80% of zinc is combined with albumin in serum, with an increase in urinary albumin levels, while serum albumin and serum zinc levels decrease in patients with advanced chronic kidney disease. Non-dialysis, chronic kidney disease (CKD) patients are also susceptible to zinc deficiency owing to an inadequate dietary intake and reduced gastrointestinal zinc absorption [6]. Previous studies indicated that poor outcomes in maintenance hemodialysis (HD) patients were attributed to zinc deficiency [7,8,9,10].

The optimal serum zinc level in HD patients remains controversial. American Society for Parenteral and Enteral Nutrition guideline suggest that trace minerals, including zinc, should be provided to critically ill patients [11]. According to European Society for Clinical Nutrition and Metabolism guideline, zinc levels should be measured for nutrition screening [12]. Japanese practical guidelines have generally proposed a serum zinc level >80 µg/dL as a normal zinc status [13]. However, a recent study on maintenance HD patients recommended a lower serum zinc range (≤78.3 µg/dL) because of the potential for copper deficiency [14]. Previous studies suggested that a relationship exists between serum zinc levels and the nutritional status [15,16]; however, limited information is currently available on this relationship in HD patients. Furthermore, although serum zinc levels may affect the outcomes of CVD and mortality, their relationship with clinical outcomes remains unclear. 

Therefore, the aims of the present study were as follows: (1) to examine the relationship between zinc and serum trace element markers or the nutritional status obtained by bioimpedance spectroscopy in incident HD patients and (2) to investigate the impact of serum zinc levels on cardiovascular events and all-cause mortality.

## 2. Materials and Methods 

### 2.1. Study Design and Subjects

**Figure 1:**
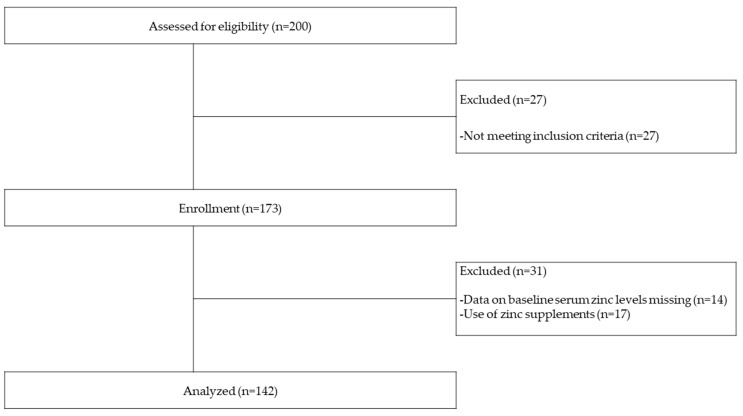
Flow of patients through the study.

This was a prospective observational study of incident HD patients from two dialysis centers for initial HD (Miyazaki Prefectural Nobeoka hospital and Chiyoda hospital) and eight dialysis facilities for maintenance HD (Ogawa Clinic, Ohnuki Clinic, Nobeoka Clinic, Naganuma Clinic, Miyata Internal Medicine Clinic, Iemura Internal Medicine Clinic, Nakamura Clinic, and National Health Insurance Takachiho Town hospital). Subjects comprised 142 patients enrolled between February 2015 and March 2019 who were followed up for a mean of 2.5 years. Inclusion criteria were incident HD patients who newly started HD for end-stage kidney disease, and giving written consent. Exclusion criteria were patients younger than 18 years of age, pregnant women, patients with acute kidney injury, and those not wishing to participate. Thirty-one patients were also excluded because of the use of zinc supplements and missing serum zinc baseline data (Figure 1).

### 2.2. General Clinical Data, Measurement of Laboratory Indices, and Body Composition

Information on physical characteristics, laboratory data, basal renal diseases, comorbidities, and medication was obtained by doctors at each dialysis center at the start of the study. Blood samples were collected in a supine position pre-HD in the first dialysis session. Serum zinc levels were measured by the atomic absorption spectrophotometry, while serum iron levels by the 2-Nitroso-5-[N-n-propyl-N-(3-sulfopropyl)amino] phenol (Nitroso-PSAP) method. Serum copper levels were measured commercially by the colorimetric method (Clinical Pathology Laboratory, Inc., Kagoshima, Japan).

Body composition was measured by bioimpedance spectroscopy using BCM™ (Body Composition Monitor^TM^, Fresenius Medical Care Japan K.K., Tokyo, Japan), which takes measurements at 50 different frequencies in the range of 5 to 1000 kHz. Electrodes were attached to the hand and foot of the non-dominant side of the body after the patient had been in a recumbent position for at least 5 min. The lean tissue index (LTI), fat tissue index (FTI), overhydration (OH), total body water (TBW), extracellular water (ECW), intracellular water (ICW), body cell mass index (BCMI), OH/ECW, and ECW/ICW were measured. The phase angle (PhA) was calculated using the following equation: PhA (degrees) = (Resistance/Reactance) × (180/π). Resistance and reactance were measured at 50 kHz. Only one BCM™ measurement at pre-HD was performed for each patient because the method had good reproducibility.

The geriatric nutritional risk index (GNRI) was calculated using the following equation developed for geriatric patients: GNRI = [14.89 × albumin (g/dL)] + [41.7 × (body weight/ideal body weight)] [17]. Body weight/ideal body weight was set to 1 when actual body weight exceeded ideal body weight. Ideal body weight was calculated from height and a body mass index (BMI) of 22 because of its validity [18].

### 2.3. Outcomes

All-cause mortality was assessed monthly by nursing staff or medical doctors during the follow-up period using questionnaires, which were examined by two authors (T.T., R.T.) where necessary. Check sheets were collected annually. Survival time was defined as the time from enrollment to individual outcomes, the data for which were collected longitudinally during the course of the study follow-up until March 2020. Cardiovascular events comprised the following: ischemic or hemorrhagic stroke, acute myocardial infarction, causes related to congestive heart failure, sudden death, or aortic aneurysm rupture. Stroke was diagnosed using typical imaging and physical findings from examinations. Acute myocardial infarction was diagnosed using typical electrocardiogram findings or elevations in myocardium-derived enzymes. Cardiac disease was confirmed based on a history of ischemic heart disease and/or congestive heart failure. Ischemic heart disease was defined as prior hospitalization or medication for angina pectoris and/or myocardial infarction. Congestive heart failure was confirmed using electrocardiography, chest radiography, or echocardiography, together with dyspnea or edema. Sudden death was judged as unexpected death in the first hour following the start of symptoms or when the patient was found dead and had been seen alive 24 h earlier.

### 2.4. Statistical Analysis

All continuous variables were tested for a normal distribution by the Kolmogorov–Smirnov test, and data were expressed as means ± standard deviation (SD) for a normal distribution or medians (interquartile range) for a non-normal distribution. Descriptive analyses were calculated to describe variables, such as patient characteristics, in the following three groups distributed based on serum zinc levels: tertile, <45, 45–59, and ≥60 µg/dL. All continuous variables were examined using a one-way analysis of variance (for a normal distribution) or Kruskal–Wallis test (for a non-normal distribution) or the χ2 test was applied for comparisons of the three groups. Spearman’s rank-order correlation analyses were used to evaluate relationships between serum zinc levels and clinical parameters. Zinc deficiency can be due to nutritional deficiency and is common in the elderly [19]. Furthermore, it may also be associated with diabetes [20] and gender [21,22]. Therefore, we investigated between the groups stratified by gender and the presence/absence of diabetes as well as in the entire study population. Crude survival in a group was assessed using a Kaplan–Meier analysis with the Log-rank test. In Cox’s regression analysis, our model included adjustments for age, sex, diabetes, body mass index, previous history of CVD, serum albumin, C-reactive protein, calcium, phosphate, intact parathyroid hormone, copper, the estimated glomerular filtration rate, PhA, OH/ECW, anti-hypertensive drugs, and zinc supplementation. All covariates were divided into categorical groups. We defined age per 10 years and others as quartile categories. Patients with a zinc level of ≥60 µg/dL were set as our reference category. All covariates conformed to the proportional hazards model using the Kaplan–Meier method and log-log plot. A multiple imputation approach using chained equations was used to account for missing covariates. All statistical analyses were performed with SPSS Statistics 26 (IBM Company, Chicago, IL, USA) and STATA version 15 (Stata Corp., College Station, Texas, TX, USA). *p* < 0.05 was considered to be significant.

### 2.5. Ethical Considerations

All subjects gave their written informed consent for inclusion before they participated in the present study. This study was conducted in accordance with the principles contained in the Declaration of Helsinki and was approved by the Miyazaki Prefectural Nobeoka Hospital Research Ethics Committee (Approval number: 20191004-3). Data collection was performed in a manner that maintained patient anonymity (UMIN000018181). Data contained no identifying personal information. The present study was conducted in accordance with Japan’s privacy protection laws; ethical guidelines for epidemiological studies published by the Ministry of Education, Science, and Culture, and the Ministry of Health, Labor, and Welfare in 2005; and the STROBE guidelines.

## 3. Results

### 3.1. Study Participants and Baseline Characteristics

**Figure 2:**
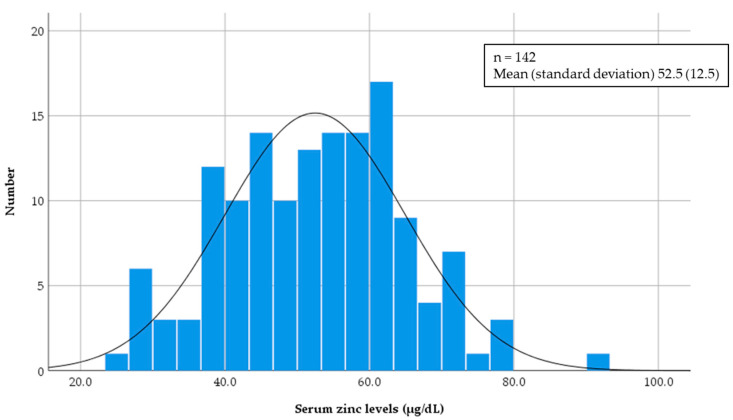
Histogram of serum zinc levels at baseline.

In total, 142 patients in the cohort study (Figure 1) were enrolled as subjects. At the initiation of HD, the mean serum zinc level (SD) was 52.5 (12.5) µg/dL and had a normal distribution (by Kolmogorov–Smirnov test, *p* = 0.20) (Figure 2). Table 1 shows baseline subject characteristics in the three groups, which were divided according to baseline serum zinc levels at the first HD. Significant differences were observed in sex, serum albumin, serum C-reactive protein, eGFR, GNRI, PhA, OH, OH/ECW, and ECW/ICW by a one-way analysis of variance, the Kruskal–Wallis test, or χ^2^ test.

### 3.2. Correlation Analysis

As shown in Table 2, serum zinc levels positively correlated with serum albumin levels, GNRI, and PhA and negatively correlated with eGFR, serum C-reactive protein, OH, OH/ECW, ECW, and E/I. On the other hand, significant differences were not observed between serum zinc levels and copper or iron levels. Following the stratification of groups by gender and the presence/absence of diabetes, serum albumin levels, GNRI, and PhA were positively correlated, while OH, OH/ECW, and ECW/ICW were negatively correlated with serum zinc levels in all groups (Figure S1 of Supplementary Materials).

### 3.3. Analysis of All-Cause Mortality and Cardiovascular Events

**Figure 3:**
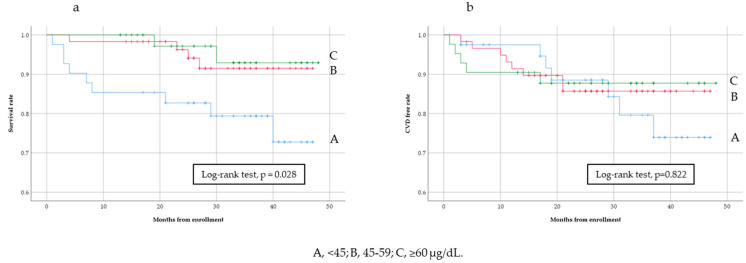
Kaplan–Meier estimates and Log rank test for all-cause mortality (**a**) and cardiovascular disease (CVD)-free (**b**) rates among baseline serum zinc levels.

During the follow-up period, there were 15 cases of all-cause mortality and 20 of cardiovascular events. Figure 3a shows that the all-cause mortality rate was significantly higher in the lowest group (<45 µg/dL) than in the other groups (Kaplan–Meier analysis, Log-rank test, *p* = 0.028). Similar results were obtained for cardiovascular events; however, the differences observed were not significant (Figure 3b, Kaplan–Meier analysis, Log-rank test, *p* = 0.822). Table 3 shows the results of unadjusted and adjusted analyses of all-cause mortality and cardiovascular events. Hazard ratios (HRs) were higher in the lowest group than in the reference group (Table 3) and increased with decreases in serum zinc levels. Similar results were obtained for cardiovascular events (Table 4).

## 4. Discussion

The results of the present baseline data analysis in this prospective observational study showed that serum zinc levels were associated with the nutritional status (serum albumin levels, GNRI, and PhA) and the fluid volume index (OH, OH/ECW, and E/I). On the other hand, serum iron and copper levels were not associated with serum zinc levels.

Patients with advanced CKD frequently have a poor nutritional status, which is associated with increased morbidity and mortality [23]. Previous studies investigated serum zinc levels and the nutritional status in maintenance HD patients. Serum zinc levels were found to positively correlate with the nutritional status in HD patients, which was assessed by measuring abdominal fat areas using computed tomography [15]. Furthermore, a previous meta-analysis revealed that zinc supplementation improved the nutritional status of maintenance HD patients [16]. GNRI has been shown to correlate with a number of nutrition-related markers and has been validated as a significant predictor of morbidity and mortality [18,24,25]. PhA is less sensitive to the influence of the body fluid status, is a useful nutritional indicator in HD patients with large fluctuations in the body fluid status [26], and may be associated with mortality [27]. These findings indicate the utility of serum zinc levels in assessments of the nutritional status, similar to other indicators. However, the negative relationship between serum zinc levels and the fluid volume index may be affected by the body fluid status. Malnutrition may result in increased fluid volume in the 3 rd space and/or the intracellular space as a result of low osmotic pressure or increased vascular permeability with inflammation [28]. In addition, intestinal edema, which may lead to malabsorption, may also form a negative cycle of nutrition status. Therefore, the body fluid status should be carefully monitored during blood sampling because the effects of dilution, due to the increase in body fluid volume, on serum zinc levels cannot be ruled out.

A previous study in patients on HD reported that zinc and copper levels were weakly, inversely correlated. However, copper deficiency may be a side effect caused by zinc supplementation [14]. In this study, a significant relationship between serum zinc and serum copper may not have been found because we excluded those who were receiving zinc preparations, unlike the previous study [14]. On the other hand, iron deficiency may cause low zinc levels, as zinc protoporphyrin is formed instead of protoporphyrin, which incorporates an atom of zinc rather than iron [29,30]. In this study, iron medication is not restricted and is given in patients who need it to be administered, thus serum iron levels were not associated with serum zinc levels.

The longitudinal study using a Kaplan–Meier analysis showed that low serum zinc levels were associated with all-cause mortality. However, in this study, the association between serum zinc levels and all-cause mortality or cardiovascular events was not clear after adjustments for potential confounders. Previous studies demonstrated that zinc intake correlated with serum zinc levels in a general population [31,32]. Non-dialysis CKD patients are susceptible to zinc deficiency owing to an inadequate dietary intake and reduced gastrointestinal zinc absorption [6]. Furthermore, a low daily intake of sodium due to salt restrictions is associated with an inadequately low intake of trace elements, including zinc [33]. Therefore, the relationship between low zinc levels and outcomes in the present study appears to reflect a poor nutritional status. Furthermore, zinc deficiency in non-dialysis CKD may be another risk factor for the development of atherosclerosis. Calcification of the abdominal aorta was previously found to be independently associated with cardiovascular events in HD and non-dialysis CKD patients as well as in a general population [34,35,36]. Furthermore, a high dietary zinc intake was associated with a reduced risk of severe abdominal aortic calcification [37]. In non-dialysis CKD patients, a higher zinc intake was associated with lower odds of severe abdominal aortic calcification following adjustments for age, sex, and race. Relationships have already been demonstrated among zinc deficiency, oxidative stress, inflammation, and the development of CVD [19,38,39]. Lobo et al. reported that a reduced antioxidant function caused by zinc depletion is possibly associated with atherosclerosis in non-dialysis CKD patients [40]. Furthermore, in HD patients, plasma zinc levels were negatively correlated to tumor necrosis factor-alpha levels and electronegative low-density lipoprotein levels [41]. In addition, zinc supplementation has been suggested to reduce the risk and progression of atherosclerosis [41,42].

The major strength of the present study is that it is the first longitudinal study to have examined the relationship between serum zinc levels and clinical outcomes in incident HD patients. However, there are potential limitations that need to be addressed. As the sample size in this cohort was small, it was not representative of the incident HD patient population, which may limit the generalizability of the present results. Serum zinc levels were measured at incident HD, and the serum status at entry did not consider variability causes by HD and over time. Furthermore, data on changes in zinc levels and zinc supplementation after initial HD were not available, thus we cannot exclude the possibility that serum zinc values are prognostic rather than etiological factors. Additionally, serum trace element levels may not be the most accurate indicator of total body stores.

## 5. Conclusions

Serum zinc levels positively correlated with the nutritional and fluid volume status in initial HD patients. Furthermore, a relationship appears to exist between serum zinc levels and all-cause mortality and cardiovascular events. Although the reference level for serum zinc levels in HD patients has not yet been established, the present results suggest that zinc plays an important role in the clinical outcomes of initial HD patients. The present results need to be confirmed in larger studies, and interventional studies are also warranted.

## Figures and Tables

**Table 1:** Baseline characteristics.

		Serum Zinc Levels (µg/dL)			
	All	<45	45–59	≥60	*p* Value *
*n*	142	41	59	42	
Age (years)	70.0 ± 11.0	71.0 ± 11.2	69.8 ± 11.0	69.2 ± 11.0	0.073
Male	84, 59.2	32, 78.0	28, 47.5	24, 57.1	0.009
Diabetes	70, 49.3	23, 56.1	30, 50.8	17, 40.5	0.346
BMI (kg/m^2^)	24.1 ± 4.5	23.5 ± 3.7	24.4 ± 4.6	24.4 ± 5.0	0.548
SBP (mmHg)	157 ± 24	155 ± 21.4	159 ± 26	155 ± 26	0.549
DBP (mmHg)	78 ± 13	77 ± 15	77 ± 12	81 ± 14	0.318
Previous history of CVD	50, 35.2	20, 51.2	16, 27.1	14, 33.3	0.079
<Lab data>					
Serum zinc (µg/dL)	52.5 ± 12.5	37.4 ± 5.4	52.8 ± 4.4	66.8 ± 6.3	<0.001
Serum copper (µg/dL)	106.5 ± 26.0	107.0 ± 30.4	106.1 ± 22.2	106.6 ± 27.0	0.985
Serum iron (µg/dL)	53 (33–77)	44 (32–67)	54 (31–79)	56 (37–92)	0.306
TSAT (%)	24.7 (16.8–35.9)	21.4 (16.3–35.1)	25.5 (15.5–34.8)	25.3 (17.8–36.8)	0.096
Serum ferritin (ng/dL)	149 (63–248)	176 (124–266)	114 (50–212)	146 (69–264)	0.069
Hemoglobin (g/dL)	8.8 ± 1.7	8.7 ± 2.5	8.6 ± 1.4	9.0 ± 1.3	0.510
Serum albumin (g/dL)	3.1 ± 0.6	2.6 ± 0.6	3.1 ± 0.5	3.4 ± 0.5	<0.001
Serum BUN (mg/dL)	84.3 ± 35.0	84.0 ± 30.8	80.3 ± 36.6	90.2 ± 36.6	0.376
Serum Cre (mg/dL)	7.9 ± 2.7	7.5 ± 3.5	8.0 ± 2.4	8.1 ± 2.2	0.619
eGFR (ml/min/1.73 m^2^)	5.7 (4.4–7.1)	6.6 (5.2–8.3)	5.3 (3.9–7.1)	5.4 (4.3–6.6)	0.024
Serum CRP (mg/dL)	0.4 (0.1–2.5)	1.6 (0.2–8.2)	0.3 (0.1–1.5)	0.3 (0.1–1.3)	0.003
Serum aCa (mg/dL)	8.9 (8.4–9.3)	8.9 (8.6–9.3)	8.8 (8.4–9.4)	8.9 (8.4–9.2)	0.652
Serum P (mg/dL)	5.7 (4.7–6.9)	5.1 (4.3–6.0)	5.9 (4.9–7.2)	5.9 (4.7–6.9)	0.069
Serum iPTH (pg/dL)	265 (154–437)	231 (125–295)	281 (158–476)	288 (151–465)	0.175
NTproBNP (pg/mL)	5084 (1740–17,508)	6743 (2700–25,443)	3296 (1629–14,078)	5344 (1317–16,656)	0.181
GNRI	85.1 (77.4–91.1)	76.7 (70.6–84.6)	85.0 (79.7–91.7)	89.4 (86.4–96.5)	<0.001
<Medication>					
Iron supplements	13, 9.2	4, 9.8	6, 10.2	3, 7.1	0.863
Phosphate binders	38, 26.8	11, 26.8	16, 27.1	11, 26.2	0.995
Anti-hypertensives	115, 81.6	32, 80.0	49, 83.1	34, 81.0	0.922
ESA	133, 93.7	38, 92.7	57, 96.6	38, 90.5	0.439
<BCM>					
PhA (degrees)	3.7 ± 1.1	3.1 ± 1.0	3.9 ± 1.0	4.1 ± 1.1	0.001
OH (liter)	4.0 ± 3.5	5.2 ± 4.3	4.1 ± 3.2	2.6 ± 2.4	0.003
OH/ECW	0.2 (0.1–0.3)	0.3 (0.2–0.4)	0.2 (0.1–0.3)	0.2 (0.1–0.3)	0.001
TBW (liter)	33.7 (28.2–37.8)	34.5 (31.0–41.0)	32.6 (26.6–36.9)	33.7 (27.9–37.5)	0.158
ECW (liter)	17.0 (14.1–19.0)	17.8 (16.2–21.2)	16.5 (13.7–19.0)	15.9 (13.8–18.5)	0.061
ICW (liter)	16.1 (13.3–19.1)	16.7 (14.5–19.0)	15.2 (12.5–18.0)	17.0 (12.9–20.0)	0.158
ECW/ICW	1.1 (0.9–1.2)	1.1 (1.0–1.2)	1.1 (0.9–1.2)	1.0 (0.9–1.1)	0.005
LTI (kg/m^2^)	13.8 ± 3.4	13.8 ± 3.3	13.5 ± 3.3	14.2 ± 3.7	0.562
FTI (kg/m^2^)	8.6 ± 4.8	7.6 ± 3.7	9.3 ± 4.9	8.7 ± 5.6	0.248
BCMI	7.7 ± 2.4	7.7 ± 2.4	7.5 ± 2.3	8.0 ± 2.6	0.585

Continuous variables are shown as means ± standard deviation for a normal distribution or medians (interquartile range) for a non-normal distribution. Categorical variables are shown as numbers and percentages. * By a one-way analysis of variance or the Kruskal–Wallis test or χ^2^ test. BMI, body mass index; SBP, systolic blood pressure; DBP, diastolic blood pressure; CVD, cardiovascular disease; TSAT, Transferrin saturation; BUN, blood urine nitrogen; eGFR, estimated glomerular filtration rate; CRP, C-reactive protein; aCa, adjusted calcium; P, phosphate; iPTH, intact parathyroid hormone; NTproBNP, *N*-terminal pro-brain natriuretic peptide; GNRI, geriatric nutritional risk index; ESA, erythropoiesis-stimulating agent; BCM, Body Composition Monitor; PhA, phase angle; OH, overhydration; ECW, extracellular water; TBW, total body water; ICW, intracellular water; LTI, lean tissue index; FTI, fat tissue index; BCMI, body cell mass index.

**Table 2:** Correlation analyses of serum zinc and clinical parameters.

	All Patients (*n* = 142)		Male (*n* = 84)		Female (*n* = 58)		Diabetic (*n* = 70)		Non-Diabetic (*n* = 72)	
	r	*p*-Value	r	*p*-Value	r	*p*-Value	r	*p*-Value	r	*p*-Value
Age (years)	−0.150	0.862	−0.130	0.238	0.217	0.102	0.064	0.597	−0.127	0.289
BMI (kg/m^2^)	0.046	0.590	0.021	0.849	0.050	0.709	0.061	0.619	0.101	0.400
SBP (mmHg)	0.027	0.751	−0.091	0.412	0.169	0.206	0.061	0.617	−0.010	0.936
DBP (mmHg)	0.118	0.161	0.123	0.267	0.168	0.207	0.010	0.932	0.152	0.204
<Lab data>										
Serum copper (µg/dL)	−0.020	0.818	−0.003	0.982	0.072	0.590	−0.220	0.860	0.036	0.763
Serum iron (µg/dL)	0.152	0.082	0.215	0.061	0.076	0.587	0.153	0.219	0.111	0.379
TSAT (%)	0.092	0.301	0.128	0.269	0.051	0.715	0.068	0.588	0.080	0.531
Serum ferritin (ng/dL)	−0.006	0.947	−0.090	0.434	0.202	0.142	−0.040	0.753	0.016	0.902
Hemoglobin (g/dL)	0.349	0.079	0.402	0.093	0.506	0.086	0.550	0.073	0.373	0.106
Serum albumin (g/dL) *	0.518	<0.001	0.557	<0.001	0.394	0.002	0.582	<0.001	0.460	<0.001
eGFR (ml/min/1.73 m^2^)	−0.251	0.003	−0.208	0.057	−0.226	0.088	−0.0178	0.140	−0.287	0.014
Serum CRP (mg/dL)	−0.353	<0.001	−0.380	<0.001	−0.157	0.238	−0.530	<0.001	−0.241	0.042
Serum aCa (mg/dL)	−0.440	0.607	0.013	0.909	−0.146	0.274	−0.092	0.450	−0.012	0.920
Serum P (mg/dL)	0.158	0.060	0.188	0.087	0.052	0.698	−0.099	0.416	0.320	0.006
Serum iPTH (pg/dL)	0.184	0.139	0.118	0.314	0.179	0.203	0.137	0.285	0.180	0.156
NTproBNP (pg/mL)	0.037	0.661	0.028	0.802	0.053	0.690	−0.063	0.607	0.162	0.175
GNRI *	0.505	<0.001	0.411	0.001	0.383	0.003	0.512	<0.001	0.481	<0.001
<BCM>										
PhA (degrees) *	0.287	0.001	0.458	0.001	0.267	0.015	0.300	0.012	0.264	0.025
OH (liter) *	−0.384	<0.001	−0.341	0.001	−0.401	0.002	−0.398	0.001	−0.342	0.006
OH/ECW *	−0.364	<0.001	−0.376	0.001	−0.444	<0.001	−0.418	<0.001	−0.330	0.005
TBW (liter)	−0.124	0.146	−0.019	0.863	−0.124	0.360	−0.129	0.287	−0.104	0.394
ECW (liter)	−0.237	0.005	−0.192	0.080	−0.152	0.254	−0.257	0.032	−0.195	0.101
ICW (liter)	0.063	0.457	0.121	0.272	0.228	0.850	0.060	0.612	0.047	0.693
ECW/ICW *	−0.287	0.001	−0.339	0.002	−0.315	0.016	−0.254	0.035	−0.281	0.017
LTI (kg/m^2^)	0.048	0.573	0.140	0.208	0.049	0.716	0.122	0.313	−0.024	0.844
FTI (kg/m^2^)	0.048	0.570	0.017	0.880	0.059	0.659	0.039	0.753	0.169	0.160
BCMI	0.053	0.532	0.136	0.221	0.046	0.731	0.129	0.285	−0.027	0.823

BMI, body mass index; SBP, systolic blood pressure; DBP, diastolic blood pressure; TSAT, Transferrin saturation; BUN, blood urine nitrogen; eGFR, estimated glomerular filtration rate; CRP, C-reactive protein; aCa, adjusted calcium; P, phosphate; iPTH, intact parathyroid hormone; NTproBNP, *N*-terminal pro-brain natriuretic peptide; GNRI, geriatric nutritional risk index; BCM, Body Composition Monitor; PhA, phase angle; OH, overhydration; ECW, extracellular water; TBW, total body water; ICW, intracellular water; LTI, lean tissue index; FTI, fat tissue index; BCMI, body cell mass index. * *p* < 0.05 in all patients and stratified by sex and diabetes.

**Table 3:** Relationship between baseline serum zinc levels and hazard ratios of all-cause mortality.

Serum Zinc Levels (µg/dL)	Number of Deaths, %	Unadjusted Model	Adjusted Model 1 *	Adjusted Model 2 **
<45	9, 22.0	4.61 (0.99–21.33)	4.23 (0.87–20.64)	6.48 (0.25–166.94)
45–59	4, 6.8	1.43 (0.26–7.79)	1.44 (0.26–7.91)	3.84 (0.21–50.94)
≥60	2, 4.8	1.00 (reference)	1.00 (reference)	1.00 (reference)

Values shown are hazard ratios (95% confidence interval). * Adjusted for age and sex. ** Adjusted for age, sex, diabetes, body mass index, previous history of cardiovascular disease (CVD), hemoglobin, serum albumin, C-reactive protein, adjusted calcium, phosphate, intact parathyroid hormone, and copper; eGFR, phase angle, OH/ECW, and the use of anti-hypertensive drugs.

**Table 4:** Relationship between baseline serum zinc levels and hazard ratios of cardiovascular events.

Serum Zinc Levels (µg/dL)	Number of Deaths, %	Unadjusted Model	Adjusted Model 1 *	Adjusted Model 2 **
<45	7, 17.1	1.40 (0.46–4.42)	1.25 (0.41–3.84)	2.47 (0.21–29.30)
45–59	8, 13.6	1.10 (0.36–3.35)	1.19 (0.37–3.84)	1.58 (0.13–18.60)
≥60	5, 11.9	1.00 (reference)	1.00 (reference)	1.00 (reference)

Values shown are hazard ratios (95% confidence interval). * Adjusted for age and sex. ** Adjusted for age, sex, diabetes, body mass index, previous history of cardiovascular disease (CVD), hemoglobin, serum albumin, C-reactive protein, adjusted calcium, phosphate, intact parathyroid hormone, and copper; eGFR, phase angle, OH/ECW, and the use of anti-hypertensive drugs.

## Supplementary Materials

The following are available online at https://www.mdpi.com/2072-6643/12/10/3187/s1, Figure S1: Relationships between serum zinc levels and serum albumin levels.
