# Supplementary material for: Association between Serum Zinc Levels and Clinical Index or the Body Composition in Incident Hemodialysis Patients
Source: Nutrients. 2020 Oct 19;12(10):3187. doi: 10.3390/nu12103187 (PMC7603268; doi:10.3390/nu12103187)
Supplement: Supplementary file 1 [file nutrients-12-03187-s001.pdf]

nutrients

MDPI

Supplementary Materials:

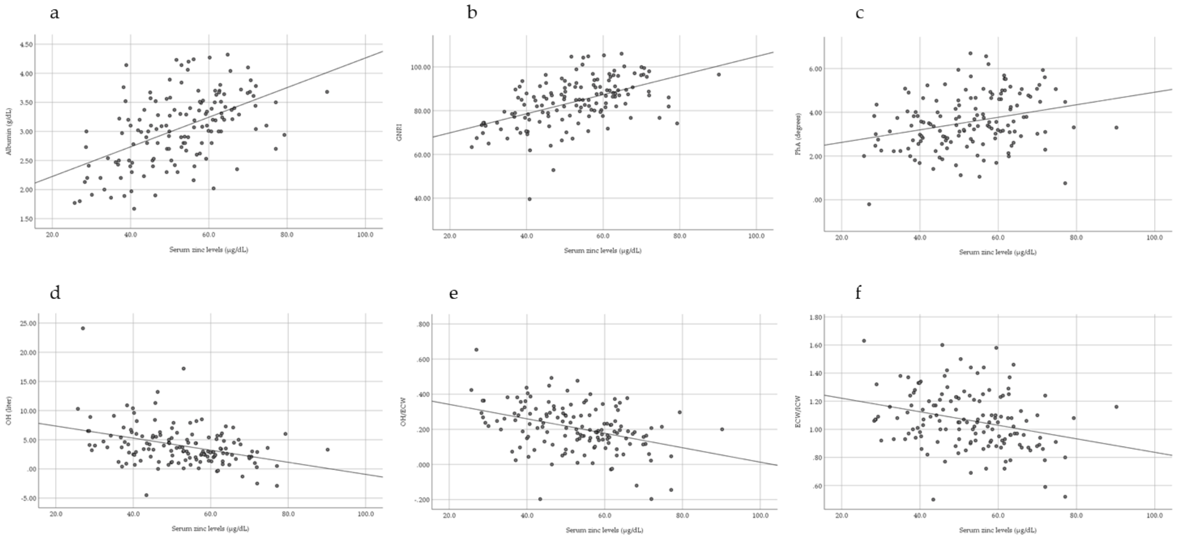

Figure S1. Relationships between serum zinc levels and serum albumin levels (a), GNRI (b), PhA (c), OH (d), OH/ECW (e), and ECW/ICW (f).
